# AKT1^low^ quiescent cancer cells in ductal carcinoma in situ of the breast

**DOI:** 10.1038/s41523-019-0105-y

**Published:** 2019-03-21

**Authors:** Sheheryar Kabraji, Xaiver Sole, Ying Huang, Clyde Bango, Dennis Sgroi, Massimo Loda, Sridhar Ramaswamy

**Affiliations:** 1 Department of Medical Oncology, Dana Farber Cancer Institute, Boston, MA 02215 USA; 2 Cancer Prevention and Control Program, Catalan Institute of Oncology (ICO), Consortium for Biomedical Research in Epidemiology and Public Health (CIBERESP), Barcelona, Spain; 3 Department of Oncologic Pathology, Dana Farber Cancer Institute, Boston, MA 02215 USA; 4 Department of Pathology, Massachusetts General Hospital, Boston, MA 02114 USA; 5 Center for Cancer Research, Massachusetts General Hospital, Boston, MA 02114 USA

## Abstract

Ductal carcinoma in situ (DCIS) of the breast precedes the development of invasive breast cancer and reflects the genomic changes and protein expression profile of invasive disease. AKT1^low^ cancer cells (QCC) are a rare, drug-tolerant, epigenetically plastic, and quiescent cancer cell subset that we previously identified at a frequency of 0.5–1% in primary breast tumors using the marker profile: AKT^low^/H3K9me2^low^/HES1^high^. Here we used quantitative immunofluorescence microscopy with computational image analysis to show that AKT1^low^ QCCs are present in DCIS from patients with and without co-existing invasive breast cancer. These data suggest that a drug-resistant, quiescent cancer cell state is present in premalignant breast lesions prior to the development of invasive disease. These findings warrant further study of whether AKT1^low^ QCCs contribute to invasive tumor development and recurrence, similar to their role in more advanced malignancy.

## Introduction

Ductal carcinoma in situ (DCIS) of the breast is a premalignant lesion that will develop into invasive breast cancer in about 20–50% of cases.^1^ DCIS reflects the genomic changes (e.g., copy number changes, point mutations) and protein expression profile (e.g., estrogen receptor (ER), HER2 receptor) of subsequent invasive disease.^2,3^ However, little is known about whether quiescent cancer cell states exist in preinvasive lesions like DCIS and might contribute to tumorigenesis.^1^ The AKT1^low^ quiescent cancer cell (QCC) is a rare, drug-tolerant, plastic, quiescent (Ki67^low^/MCM2^low^) cancer cell state that contributes to tumor growth in xenograft models.^4–7^ We previously showed that QCCs exist in clusters at a frequency of 0.5–1% in primary breast tumors and metastases using the marker profile: AKT^low^/H3K9me2^low^/HES1^high^.^7,8^ We also found that AKT1^low^ QCCs persist after neoadjuvant chemotherapy in patients with triple negative breast cancer at primary and metastatic sites and, thus, may contribute to breast cancer growth and recurrence.^7,8^

Here we used quantitative immunofluorescent microscopy with computational image analysis to show that AKT1^low^ QCCs are present in DCIS from patients with and without co-existing invasive breast cancer.

## Results

**Fig. 1:**
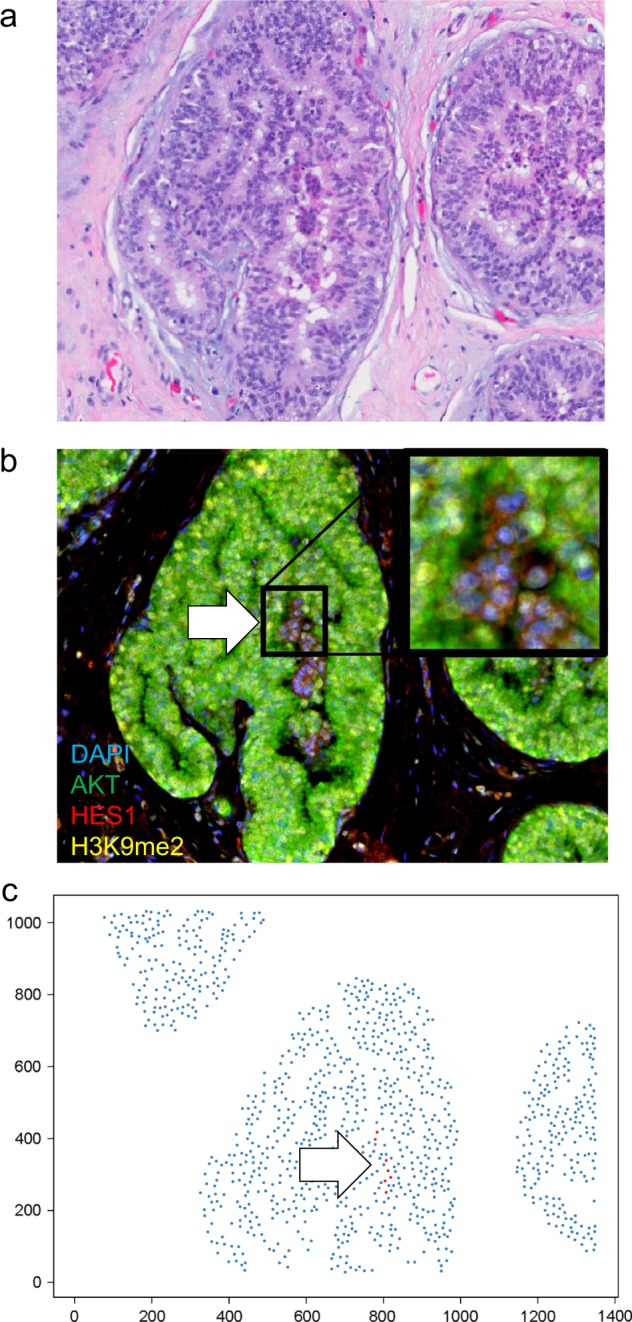
AKT1^low^ QCCs are found within DCIS. **a** Hematoxylin and eosin stained section showing a representative area of ductal carcinoma in situ; **b** Multiplex immunofluorescence of DCIS tissue shown in **a** with AKT1^low^ QCC cluster marked by a white arrow and the marker profile: AKT^low^ (green), H3K9me2^low^ (yellow) and HES1^high^ (red); inset image is enlarged to show QCC marker detail. **c** Digital tumor map of DCIS tissue shown in **a** and **b** with AKT1^low^ QCCs shown in red and proliferating cancer cells in blue. Images were taken at ×20

**Fig. 2:**
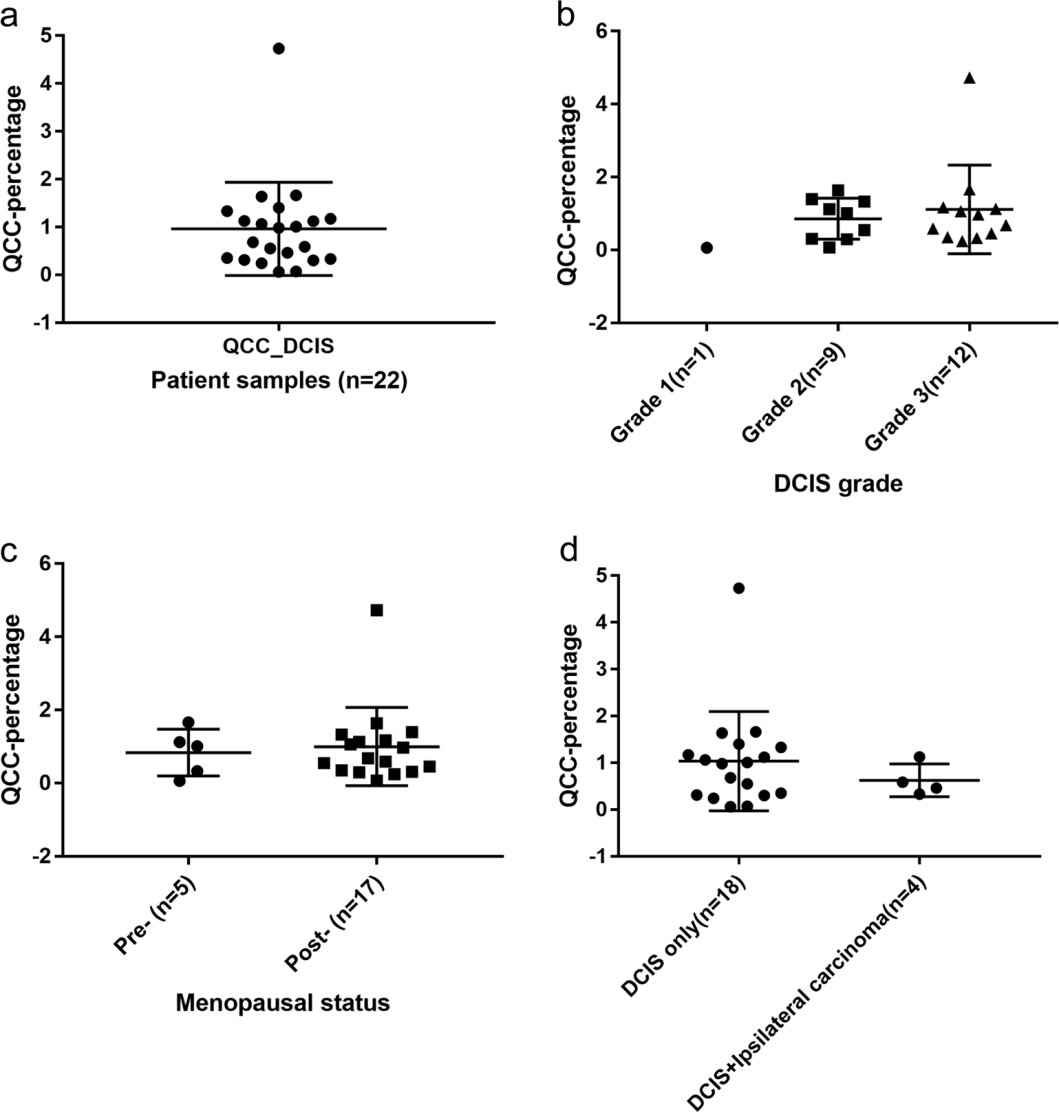
AKT1^low^ QCCs are rare and QCC-P in DCIS does not differ by grade, patient menopausal status or presence of invasive carcinoma. **a** QCCs were identified in all DCIS samples (22/22) at a mean percentage of 0.95% ± 0.93 per patient sample (one section), similar to the frequency in invasive breast cancer. **b** QCC-P did not significantly differ by DCIS grade (1, 2 or 3, *p* = 0.56); patient menopausal status (pre- vs. post-, *p* = 0.75) or **c** presence of concurrent ipsilateral invasive carcinoma (*n* = 18 vs. 4; *p* = 0.51) (**d**). Error bars represent mean ± SD

Twenty-two cases of DCIS were analyzed for AKT1^low^ QCCs. 21 cases (96%) were ER+ HER2− and 1 case was ER+ HER2 +. QCCs existed in heterogeneously distributed clusters, as we had found in invasive disease^8^ (Fig. 1a–c). QCCs were identified in all DCIS samples (22/22) at a mean percentage (QCC-P) of 0.95% ± 0.93 per patient sample (one section), similar to the frequency in invasive breast cancer (Fig. 2a).^7,8^ Given the small number of samples, it was not possible to test an association between ER or HER2 receptor status and QCC-P. QCC-P did not significantly differ by DCIS grade (1, 2 or 3; *p* = 0.56, Fig. 2b), patient menopausal status (pre- vs. post-; *p* = 0.75, Fig. 2c) or presence of concurrent ipsilateral invasive carcinoma (*n* = 18 vs. 4; *p* = 0.51, Fig. 2d). Given the short follow up time, no patient had developed recurrent invasive carcinoma.

## Discussion

We identified AKT1^low^ QCCs in patients with premalignant DCIS at a similar frequency to that found in invasive breast cancer.^8^ In addition, the frequency of QCCs was not different between the clinical or pathologic groups we analyzed. This suggests that, similar to genetic changes,^2^ quiescent cancer cell states previously identified in invasive disease may also be found in DCIS. A population of CD44^+^/ALDH1^+^/Ki67^−^ cancer ‘stem cells’ has been previously described in patients with DCIS at a frequency of 0.36–15% using triple immunohistochemistry.^9^ Using flow cytometry, the same group found the frequency of CD44+/CD24−/CK+/CD45− cells in DCIS ranges from 2.6–18%.^10^ However, Ki67^low^, AKT1^low^ QCCs do not clearly express the marker profile ascribed to cancer ‘stem cells’ e.g., CD44^high^/CD24^low 4^. This suggests that there are likely multiple populations of quiescent cancer cells in DCIS but the morphologic and functional overlap between them remains to be elucidated. These findings warrant further study of how AKT1^low^ QCCs contribute to invasive tumor development and recurrence, particularly in the setting of pre-existing DCIS.

## Methods

### Patient cohort

We reviewed medical records for 80 women diagnosed with DCIS between 2015 and 2016 at Massachusetts General Hospital, under an IRB-approved discarded tissue protocol (2009P002302), for which informed consent from participants was not required. Twenty-two patients had sufficient tissue available for analysis. DCIS was confirmed by pathologic review. 4 um thick formalin-fixed paraffin embedded sections (one per patient) underwent analysis for QCCs using quantitative immunofluorescence microscopy, as previously described.^8^

### Semi-automated immunofluorescence staining and imaging

Briefly, tissue sections underwent semi-automated sequential labelling for pan-AKT at 1:3000 (CST, 4691S), HES1 at 1:1000 (EMD Millipore, AB5702) and H3K9me2 at 1:150 (Abcam, ab1220) using tyramide-signal amplifying immunofluorescence (Perkin Elmer) on an automated staining platform (Bond, Leica). Images were acquired on a semi-automated confocal microscope (Vectra, Perkin Elmer) and tissue and cell masks were applied to identify areas of DCIS, using InForm software (Perkin Elmer). AKT1^low^ QCCs were determined using prespecified fluorescence intensity thresholds, as previously described.^8^ Specifically, a cancer cell was classified as an AKT1^low^ QCC if the HES1 fluorescence intensity for that cell fell in the top 75% percentile of cancer cells for that section and the AKT and H3K9me2 fluorescence intensities for that cell fell in the bottom 25% percentile of cancer cells for that section. QCC percentage for each patient tissue section was determined and digital tumor maps were generated using R software (R Foundation).

### Determining image sample size

To be 99% confident of detecting QCCs in each section at a prevalence (P) of 1% of cancer cells, we calculated that (assuming precision of 0.00625 and Z of 2.58) we would need to image at least 1688 cancer cells per patient section.^11^ We imaged 718 ± 549 (10–3162) cancer cells per x20 field and an average of 29 (4–120) x20 fields were imaged per patient section. Thus, on average, we imaged 20,822 cancer cells per patient section and are 99% confident of detecting QCCs in each tissue section with a precision of 0.625%. Values are shown as mean ± standard deviation. Range shown is maximum and minimum. Differences in groups were determined by unpaired *t*-test or one-way ANOVA and *p* < 0.05 was considered significant using Graphpad Prism (v. 7).

### Code availability

Code used for calculating QCC-P and generating digital tumor maps is available on request.

## Data Availability

All images and raw QCC counts available on request. All other data are available from the authors.
